# New Insights Into the Long Non-coding RNA SRA: Physiological Functions and Mechanisms of Action

**DOI:** 10.3389/fmed.2018.00244

**Published:** 2018-09-06

**Authors:** Liang Sheng, Lan Ye, Dong Zhang, William P. Cawthorn, Bin Xu

**Affiliations:** ^1^Department of Pharmacology, School of Basic Medical Science, Nanjing Medical University, Nanjing, China; ^2^Neuroprotective Drug Discovery Key Laboratory of Nanjing Medical University, Nanjing, China; ^3^State Key Laboratory of Reproductive Medicine, Nanjing Medical University, Nanjing, China; ^4^University/British Heart Foundation Centre for Cardiovascular Science, The Queen's Medical Research Institute, University of Edinburgh, Edinburgh, United Kingdom; ^5^Division of Metabolism, Endocrinology and Diabetes, Department of Internal Medicine, University of Michigan Medical Center Ann Arbor, MI, United States

**Keywords:** long non-coding RNA (lncRNA), steroid receptor RNA activator (SRA), physiological functions, regulation of gene expression, differentiation

## Abstract

Long non-coding RNAs (lncRNA) are emerging as new genetic/epigenetic regulators that can impact almost all physiological functions. Here, we focus on the long non-coding steroid receptor RNA activator (SRA), including new insights into its effects on gene expression, the cell cycle, and differentiation; how these relate to physiology and disease; and the mechanisms underlying these effects. We discuss how SRA acts as an RNA coactivator in nuclear receptor signaling; its effects on steroidogenesis, adipogenesis, and myocyte differentiation; the impact on breast and prostate cancer tumorigenesis; and, finally, its ability to modulate hepatic steatosis through several signaling pathways. Genome-wide analysis reveals that SRA regulates hundreds of target genes in adipocytes and breast cancer cells and binds to thousands of genomic sites in human pluripotent stem cells. Recent studies indicate that SRA acts as a molecular scaffold and forms networks with numerous coregulators and chromatin-modifying regulators in both activating and repressive complexes. We discuss how modifications to SRA's unique stem-loop secondary structure are important for SRA function, and highlight the various SRA isoforms and mutations that have clinical implications. Finally, we discuss the future directions for better understanding the molecular mechanisms of SRA action and how this might lead to new diagnostic and therapeutic approaches.

## Introduction

Recent analysis of numerous high-throughput mammalian genomic platforms suggests that protein-coding genes constitute only 2% of the genome, while many non-coding regulatory elements are transcribed into non-coding RNA (ncRNA) (1–6). Increasing numbers of recent studies have further revealed the importance of ncRNAs in the regulation of almost all biological processes, including development, differentiation, metabolism, and disease pathogenesis (7–10). Non-coding RNAs include a diverse group of transcripts, such as ribosomal RNA, transfer RNA, small RNA, small nuclear RNA, small nucleolar RNA, regulatory ncRNAs, and other ncRNAs that are yet to be fully characterized (11, 12). Regulatory ncRNAs can be defined according to their size as small ncRNAs (<200 nt), such as micro RNA (miRNAs), endogenous small interfering RNAs (siRNA), and piwi-interacting RNA (piRNA); and long ncRNA (lncRNAs), which are >200 nt in length. Small ncRNAs have functions that include repressing target RNAs, and these have been extensively studied. In contrast, lncRNAs have been recently recognized to regulate gene expression through a range of mechanisms, such as transcriptional regulation, modulation of chromatin modification, and even organizing factors that impact nuclear shape and structure (7, 13). So far, the number of new and putative functional lncRNAs has greatly expanded, with over 16,000 and 8,000 lncRNAs annotated in the human and mouse genomes, respectively (14). Based on their locations to nearby protein-coding genes, lncRNAs can be classified into different categories, including sense, antisense, divergent, and intergenic lncRNAs (15). Most lncRNAs are transcribed by RNA polymerase II and they are 5′ capped, polyadenylated, and spliced (16, 17). Moreover, the expression of these lncRNAs is remarkably tissue-specific and is highly regulated during development and in response to physiological signals (18–22).

**Figure 1:**
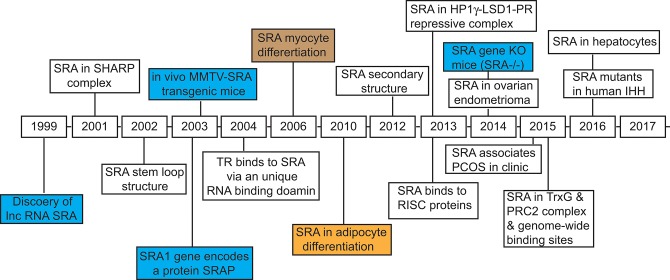
Timeline of discovery of steroid receptor RNA activator, SRA, and the identification of its biological functions. The key dates are indicated for the discovery of lncRNA SRA, and characterization of its structure and physiological functions in regulating gene expression, cell differentiation, and development of disease.

One notable lncRNA is the steroid receptor RNA activator (SRA), which is encoded by the *SRA1* gene. SRA was initially identified in 1999 by a yeast two-hybrid assay in a human B-lymphocyte library, in which the activation function 1 domain (AF-1) of progesterone receptor (PR) was used as bait (23) (Figure 1). SRA is intergenic and has a core sequence that in humans is 687 bp in length. In addition, SRA was the first lncRNA shown to act as a coactivator of steroid receptors (SRs), including the androgen receptor (AR), estrogen receptor (ER), glucocorticoid receptor (GR), and PR (23). Subsequent studies revealed that SRA also functions as an RNA coactivator for other type I and type II nuclear receptors (NRs), as well as the transcription factor MyoD. Through these molecular interactions and other pathways, SRA can impact numerous physiological and pathological processes. For example, SRA has been reported to regulate mammary gland development, myocyte, and adipocyte differentiation, steroidogenesis, tumorigenesis, hepatic steatosis, and stem cell function; indeed, aberrant SRA expression and recent mutant variants have been identified in several clinical samples (24–28). Thus, SRA is implicated in various physiological contexts and may also be relevant to pathogenesis of diverse diseases.

### The bifunctional nature of SRA1: lncRNA (SRA) vs. SRA protein (SRAP)

An important consideration relates to the bifunctional nature of the *SRA1* locus. The *SRA1* gene is unique in that it encodes not only RNA transcripts (lncSRAs) of different lengths (23, 29) but also produces, by alternative splicing, an mRNA that encodes a 236/237 amino acid protein (30, 31) (Figure 1). To avoid confusion, herein we refer to the gene as *SRA1*, the lncRNA as SRA, and the protein as SRAP.

Both SRA and SRAP have biological functions (32) although, compared to SRA, the physiological function of SRAP has been less studied. Nevertheless, *in vitro* data reveals that SRAP interacts with some transcription factors and may co-activate or repress their activity depending on the cellular context (33–35). For example, recent ChIP-on-chip arrays identified a long list of SRAP-binding partners (including the steroid receptors ERα and GR) and regions of SRAP-binding sites genome-wide (35). These studies indicate that SRAP recruits to target promoters and acts as transcriptional repressor. Recently described crystal structures have further revealed that both mouse SRAP (residues 87–216) and human SRAP (residues 105–215) include a five-helix X bundle structure (36, 37) and are structurally similar to the carboxy-terminal domain of the yeast protein PRP18, a splicing factor involved in interaction with RNA and pre-mRNA splicing (38–40). Thus, another possibility is that SRAP modulates splicing. In addition, phosphorylation of SRAP may be involved in cell cycle regulation (41). Thus, SRAP is emerging as a further means through which *SRA1* can exert diverse downstream effects.

A key implication of this bifunctionality is that one must be cautious when interpreting the results of knockdown or knockout of the endogenous *SRA1* gene. For example, siRNA- or shRNA-mediated knockdown may simultaneously deplete both SRA and SRAP. Indeed, in our recently generated *Sra1*^−/−^ mice we found that, in the tissues examined, both SRA and SRAP are lost (42). Consequently, in such systems one cannot conclude if the functional change is due to the loss of either SRA or SRAP only, or to the loss of both factors. In contrast, overexpression systems have been able to mostly overcome this issue: use of the pSCT-SRA plasmid allows for expression of SRA but not SRAP, because this plasmid contains only the SRA core sequence (23). Conversely, use of an SRAP plasmid allows expression of SRAP but not SRA, because this plasmid contains silent mutations in the major SRA stem loops and thereby causes defective SRA activity (33, 43, 44). Even so, one cannot rule out the possible roles of other stem loops and sub-structural domains of SRA, as identified in the full crystal structure of SRA (45).

While these issues are essential to consider, studies to date have focused more on SRA than on SRAP. Therefore, in this review we will prioritize discussion of the lncRNA SRA, including its molecular structure, impact on physiological processes, mechanisms of action, and relevance to disease. However, the functions and implications of SRAP will also be discussed in cases where there is sufficient data to provide further insights.

## SRA structure, evolution, and isoforms

### SRA structure

Understanding the nature of SRA structure, and how this is affected by sequence variation, is the molecular basis on which to dissect its diverse functions. Lanz and colleagues generated the secondary structure of SRA using phylogenetics, free energy calculations, and a computational MFOLD program (43, 46). By site-directed mutagenesis and functional assays they determined that the distinct RNA motifs of SRA are important for its coactivation activity (43). These motifs include 11 topological substructures (STR 1–11) that are mainly located in the stem-loop regions of the core sequence of human and mouse SRA. Among these STRs, five motifs (STRs 1, 7, 9, 10, and 11) contribute in a cooperative way to the overall coactivation. Consequently, co-transfection of STR1 and STR7 variants with silent mutations, which impair the SRA secondary structure without changing the deduced amino acid sequence, almost totally abolishes SRA coactivation of PR-mediated transcription (43). Furthermore, a recent study combined chemical probing (SHAPE, DMS, In-line, or RNase V1) with covariance analysis across multiple species, which revealed that full-length human SRA consists of four domains (I–IV) with 25 helices and a variety of secondary structure elements (45). Importantly, the secondary structures determined by these chemical probing studies (45) are consistent with the previously characterized functional substructure motifs (STRs) (43). For example, RNA motifs STR1 and STR7 are consistent with the secondary structural model of helices H2 and H13 (45), respectively. It is interesting that the previous STR7 region (now H13) is described as a “hot spot” of protein-RNA interactions and is required to interact with the SRA-associated proteins SHARP and SLIRP (47, 48).

### SRA1 gene evolution

The conservation of the *SRA1* gene has been analyzed in terms of its protein product, SRAP, in all Chordata or certain vertebrates and species from lower taxa, with the most conserved amino acids defined as two distinct N- and C-terminal domains (26, 36). Importantly, by using either ENCODE sequences or Ensemble data, and comparing the secondary structures of SRA RNA across vertebrates, it was revealed that evolutionary pressure preserves the RNA structural core in domains III and IV of SRA, rather than preserving the structure of SRAP itself (45, 49). This suggests that the primary functions of *SRA1* predominantly relate to its lncRNA product, rather than those mediated via the SRAP protein.

### SRA isoforms

Studies in HeLa cells initially identified three isoforms of human SRA with the same core sequence but different 5′ and 3′ ends (23). Subsequent work indicated that alternative splicing led to the generation of coding and various noncoding SRA isoforms in breast tissues, including the intron-1-containing SRA that disrupts the SRAP open reading frame (29). A recent study analyzed the ability of SRA isoforms to modulate activity of the ETS2 transcription factor, and further validated the existence of these different isoforms *in vivo* using 5′ RACE PCR. This revealed that, *in vivo*, 35% of SRA transcripts are SRAP-coding while 63% are non-coding isoforms that produce SRA only (33). The majority of SRA-only lncRNAs consist of differential splicing of intron 1 (29, 33) or deletion of exon 3 (50), both causing frame shifts that disrupt the ability to produce SRAP. Exons 4 and 5 were present in more than 99.7% of SRA sequences, whilst exon 2 or intron 1 were present in 86 or 33%, respectively, of total transcripts (33). The balance between fully spliced and intron-1-containing SRA RNAs varies among different breast tumors that have distinct carcinogenic properties. In particular, intron-1-containing SRA transcripts are enriched in tumors that have higher progesterone receptor content (51). This suggests possible links between SRA structure and tumorigenesis. However, before discussing the pathological implications of SRA, it is worth first considering its functions in normal physiology and development.

## Physiological function of SRA

**Figure 2:**
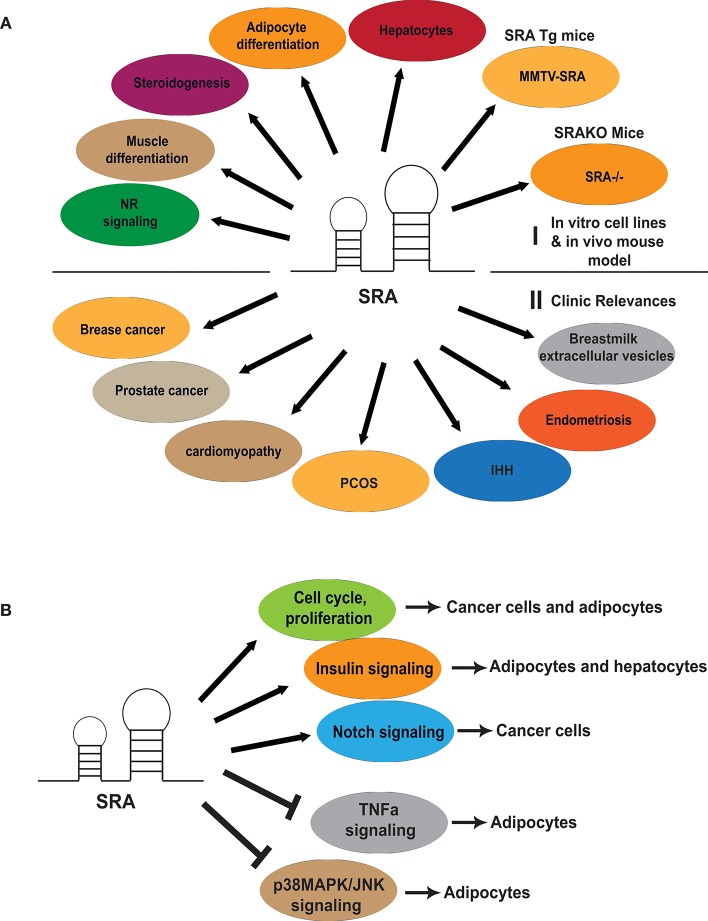
Diverse physiological functions and clinical relevance of SRA and its role in regulating cell cycle and signaling pathways. **(A)** An array of SRA functions related to regulation of myocyte and adipocyte differentiation, steroidogenesis and hepatocyte function. SRA transgene (MMTV-SRA) expression in mice led to aberrant mammary gland development, while SRA gene knockout (SRA^−/−^) in mice protected them from diet-induced obesity. SRA is also involved in breast and prostate cancer, cardiomyopathy, and reproductive disorders including polycystic ovary syndrome (PCOS), Normosmic idiopathic hypogonadotroic hypogonadism (IHH), Endometriosis. Perhaps related to its putative developmental and reproductive health functions, SRA is also expressed in extracellular vesicles (EVs) of human breastmilk. **(B)** SRA regulates cell cycle, proliferation and several signaling pathways in cancer cells and adipocytes.

Many recent studies have revealed that SRA has diverse physiological functions in development and differentiation (Figure 2A). SRA can exist in both coactivator and corepressor complexes and has been linked to regulation of NR signaling, steroidogenesis, mesenchymal fate regulation, and other physiological processes. These aspects of SRA biology are discussed further below.

### SRA regulates NR signaling

Human SRA was characterized as an RNA coactivator enhancing transactivation of steroid receptors in an AF-1 domain- and/or ligand-dependent manner (23, 52, 53). In human cell lines and *Xenopus* oocytes, human SRA was shown to form a complex with p160/SRC-1 proteins, rather than binding directly to steroid receptors (23); however, recent studies based on filter-binding assays indicate that, in human cell lines, PR directly binds to SRA in the absence of hormone (54) and that SRA also directly interacts with the N-terminal domain (NTD) of ERα and AR (55). In addition to its interaction with steroid receptors, we have further shown that human SRA physically binds to and enhances transcriptional activities of a subset of non-steroid nuclear hormone receptors, including murine thyroid hormone receptors (TRs) and steroidogenic factor 1 (SF-1), and human peroxisome proliferator-activated receptor-gamma (PPARγ). These interactions are via a unique RNA-binding domain present in the hinge regions of TR and SF-1 and in the N-terminal half of PPARγ (56–59). As we describe further below, these SRA-NR interactions are relevant to SRA's regulation of SF-1-mediated steroidogenesis and PPARγ-mediated adipocyte differentiation. In addition, mouse or human SRA enhances retinotic acid receptor (RAR)-mediated transcription (60). Thus, SRA from mice or humans can coactivate numerous NRs and regulate their physiological signaling through an NR-SRA axis.

### SRA in steroidogenesis

The NR SF-1 is essential for adrenal development and steroidogenesis. The atypical orphan NR Dax-1 binds to and represses SF-1 target genes; however, loss-of-function mutations of Dax-1 also cause adrenal hypoplasia, suggesting that Dax-1 may function as an SF-1 coactivator in some circumstances. Indeed, in Y1 mouse adrenocortical cells we recently demonstrated that both mouse SF-1 and Dax-1 bind to human SRA (59). Here, the coactivation of SF-1 by Dax-1 is abolished by SRA knockdown, leading to reduced downstream target gene expressions of steroidogenic acute regulator protein (StAR) and melanocortin 2 receptor (Mc2R) (59). Furthermore, we have shown that SRA is expressed in mouse embryonic stem cells (mES) and that Dax-1 augmentation of liver receptor homolog 1 (LRH-1)-mediated Oct4 activation is dependent upon SRA expression (61). These findings reveal a crucial function of SRA in steroidogenesis and adrenal biology.

### SRA in muscle differentiation

Human SRA is expressed at high levels in skeletal muscle (23), suggesting possible roles in muscle development and/or function. Consistent with this, Caretti and colleagues first indicated that SRA increases the activity of MyoD, the master regulator of muscle differentiation, and promotes muscle differentiation through its associations with the DEAD box-containing and RNA helicase coregulators, p68 and p72 (62). In addition, recent studies demonstrated that the role of SRA in promoting MyoD activity and muscle differentiation is counteracted by the association of SRAP (33). However, this latter observation was challenged by a recent study (37), underscoring the need for additional research to better clarify the specific role of SRA and SRAP in muscle cell differentiation.

### SRA in adipogenesis

SRA expression is two-fold higher in mouse adipocytes than in preadipocytes (56), based on which we investigated if, as for muscle differentiation, SRA might also modulate adipogenesis. We discovered a novel function of SRA as a stimulator of mouse adipocyte differentiation via directly binding to and promoting the transcriptional activity of PPARγ (56), the master regulator of adipogenesis. Notably, this is the first example of lncRNA function in adipogenesis. Microarray analysis reveals hundreds of SRA-responsive genes in mouse adipocytes themselves, which suggests further functions of SRA in these fully differentiated cells. We further revealed that SRA promotes adipogenesis and regulates insulin sensitivity in mouse adipocytes not only by acting as coactivator for PPARγ, but also via multiple additional mechanisms. These include promoting S-phase entry during the mitotic clonal expansion phase of adipogenesis; controlling cell cycle gene expression; inhibiting the expression of adipocyte-related inflammatory genes; and suppressing TNFα-induced phosphorylation of c-Jun NH2-termianl kinase, which is implicated in the development of insulin resistance in mouse adipose tissue (56). In addition, in the mouse bipotential ST2 mesenchymal cell line, SRA can also regulate adipogenesis by inhibiting phosphorylation of p38/JNK during the early stages of adipogenesis, as well as stimulating insulin receptor gene expression and downstream signaling (44).

### SRA expression in human breastmilk

The above observations underscore the function of SRA in regulation of cell fate. Development is regulated not only at this cell-fate level, but also through developmental programming that occurs *in utero* and perinatally. The latter is strongly influenced by adequate nutrition, which, in mammals, is related to breastmilk availability. Indeed, breastmilk contains nutrients, milk fat globules, hormones, growth factors, immune component cells, antibodies, cytokines, and extracellular vesicles (EVs), and plays an important role in infant development (63, 64). Recent studies suggest an unexpected role of SRA and other lncRNAs in these aspects of development. EVs are small double-lipid membrane vesicles found in a number of body fluids including breastmilk, and regulate cell-cell communications through specific interaction with target recipient cells (65, 66). A recent study has revealed that a subset of lncRNAs, including SRA1, GAS5, and CRNED, are expressed in EVs of human breastmilk (67). The implications of this remain unknown, and therefore future studies will be crucial to reveal the functional role of SRA in breastmilk; however, this observation suggests that the ability of SRA to impact development may extend beyond regulation of cell fate, including possible influences on mother-to-child signaling.

### *In vivo* functions of SRA

Lanz and colleagues generated MMTV-SRA transgenic mice, which overexpress a 1.3 kb human SRA under the control of the mouse mammary tumor virus (MMTV) promoter (68). These MMTV-SRA transgenic mice have abnormal mammary gland development characterized by ductal ectasia, acinar hyperplasia, and intraductal proliferation of luminal epithelial cells, but accompanied by increased apoptosis in the stratified epithelia. ER is the major proliferative driver in breast cancer, and SRA-mediated coactivation of the ER contributes to the increased luminal cell mitosis during mammary gland development of these transgenic mice. In addition, both male and female MMTV-SRA transgenic mice have reduced fertility, probably due to the impact of SRA on activity of sex steroid receptors.

We have recently revealed that, in mice, SRA is expressed most highly in white and brown adipose tissues compared to other tissues (42). This underscores SRA's potential function in adipose tissue formation. To further elucidate SRA function *in vivo*, the first mouse model of global SRA gene knockout (SRA^−/−^) was generated (42). Consistent with the pro-adipogenic function of SRA *in vitro*, these SRA^−/−^ mice have phenotypes of resistance to high-fat diet-induced obesity, with decreased fat mass and increased lean mass; decreased adipocyte gene expression; reduced fatty liver; and improved glucose tolerance. These data are the first in the field to indicate a functional role of lncRNA in adipose tissue biology and glucose homeostasis *in vivo*. This putative role of SRA in metabolic health is discussed further below (see section SRA, Obesity, and Fatty Liver).

## Molecular mechanisms of SRA action

**Figure 3:**
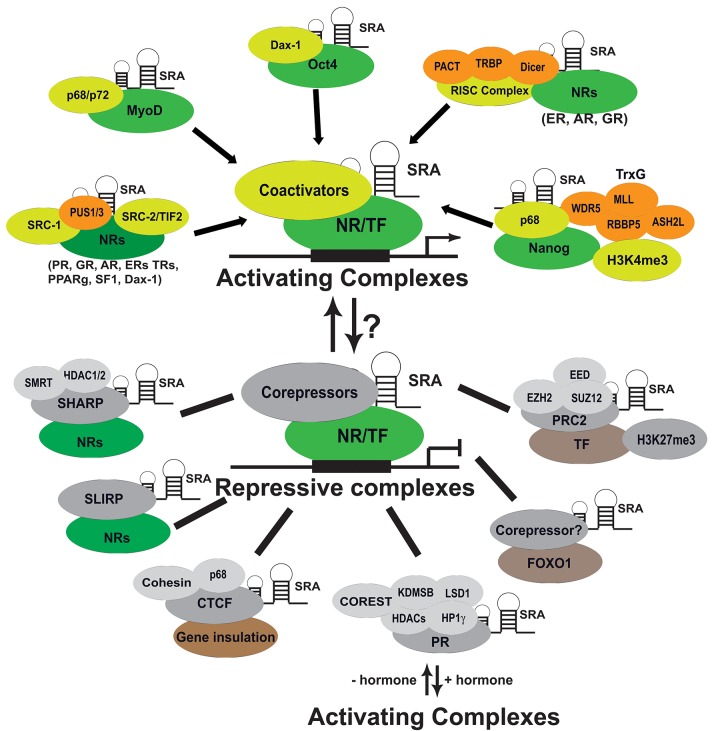
Molecular mechanism of SRA action. SRA exerts its diverse biological functions and roles in the development of cancers and other diseases through interacting with protein partners in both activating and repressive complexes. SRA also regulates the activity of transcription factors including nuclear receptors (NRs), MyoD, Oct4, and FOXO1; coregulators; and components of chromatin modification factors. PUS, pseudouridine synthase; RISC, RNA-induced silencing complex; Trxn, trithorax complex; SHARP, the SMRT/HDAC1 associated repressor protein; SLIRP, SRA stem-loop interacting RNA binding protein; CTCF, CCCTC-binding factor; HP1γ, heterochromatin protein 1γ; and LSD1, lysine-specific demethylase 1; PRC2, polycomb repressive complex 2.

We have described that SRA has diverse physiological functions in regulation of NR signaling, myocyte, and adipocyte differentiation. SRA is also implicated in the pathogenesis of breast and prostate cancers, as we discuss later in this review. Given these diverse functions, it is perhaps not surprising that SRA exerts these effects via various mechanisms and signaling pathways (Figures 2, 3).

### SRA functions as a scaffold

As described above, SRA regulates NR transcriptional activities through directly or indirectly interacting with NRs (Table 1 and Figure 3). SRA also interacts with other TFs, such as MyoD (62), Oct4 (61), Nanog (73), and FOXO1 (74); and forms a complex with coregulators of NRs and TFs, such as SRC-1 (23), P68 (62, 69), PUS1/3 (71, 75), components of RISC complex proteins (PACT, TRBP, Dicer, and Argonaut2) (72), and repressive coregulatory proteins SHARP (48) and SLIRP (47). This suggests that SRA works in concert with these interacting partners to regulate target gene expression. We describe below how SRA also binds to the components of chromatin modification complexes to regulate gene expression (Table 1 and Figure 3). Thus, SRA functions as an RNA scaffold to regulate transcription by recruitment of various regulatory molecules to transcriptional coactivator or corepressor complexes. In addition, STR7, one of the functional stem-loop substructures of SRA, is important for SRA's ability to recruit its interacting protein partners SLIRP and SHARP to DNA promoter sites (47). In contrast, the STR1 substructure mediates SRA binding to PACT, TRBP, and PKR, thereby regulating their recruitment to target promoters (72).

**Table 1:** Protein partners of lncRNA SRA.

**Proteins**	**Direct interaction**	**Complex formation**	**Functions**	**References**
PR		x	Enhances PR transactivation	(23, 54)
AR		x	Enhances AR transactivation	(23, 55)
GR		x	Enhances GR transactivation	(23)
ER		x	Enhances ER transactivation	(23, 55)
TR	x		Enhances TR transactivation	(57, 58)
RAR		x	Enhances RAR transactivation	(60)
MyoD		x	Enhances MyoD transactivation	(62)
SF-1	x		Enhances SF-1 transactivation	(59)
Dax-1	x		SRA/Dax-1 play in concert for SF-1 transactivation	(59)
PPARγ	x		Coactivator of PPARγ	(56)
SRC-1		x	Forms a ribonucleoprotein for coactivation	(23)
P68	x		Forms complexes with MyoD & component of chromatin	(62, 69, 70)
P72	x		Forms complexes with MyoD	(62)
Pus1p	x		Plays in concert for NR coactivation	(60, 71)
Pus3p	x		Plays in concert for NR coactivation	(71)
CTCF		x	Increases CTCF insulator activity	(69)
SHARP	x		SHARP represses SRA's coactivation to NR	(48)
SLIRP	x		Recruits SLIRP to binding sites	(47)
HP1γ	x		Forms a complex with PR-HP1γ-LSD1 for repression	(54)
LSD1	x		Forms a complex with PR-HP1γ-LSD1 for repression	(54)
PACT	x		Forms a complex to promote NR activity	(72)
TRBP	x		Forms a complex to promote NR activity	(72)
Dicer	x		Forms a complex to promote NR activity	(72)
Argonaute2	x		Forms a complex to promote NR activity	(72)
NANOG	x		Enhance NANOG transcriptional activity	(73)
WDR5	x		Forms TrxG complex for activation	(73)
MLL1	x		Forms TrxG complex for activation	(73)
MLL2	x		Forms TrxG complex for activation	(73)
EZH2	x		Forms PRC2 complex for repression	(70, 73)
RBBP5	x		Forms TrxG complex for activation	(73)
ASH2L	x		Forms TrxG complex for activation	(73)
EED	x		Forms PRC2 complex for repression	(73)
SUZ12	x		Forms PRC2 complex for repression	(73)
OCT4		x	Enhances OCT4 transcriptional activity	(61)
FOXO1		x	Represses FOXO1 transcriptional activity	(74)

### SRA post-transcriptional modifications

Zhao and colleagues demonstrated that a pseudouridine synthase (PUS), mPus1p, can function as a coactivator for mouse retinoic acid receptor gamma (mRARγ) and other nuclear receptors (60). They further revealed that mPus1p pseudouridylates SRA, and that co-transfection of mPus1p and SRA cooperatively enhances mRARγ-mediated transcription. mPus3p also pseudouridylates SRA, although it may target different positions of SRA than those targeted by mPus1p (71). An additional study found that uridine at 206 of SRA (U206) is the common target position for both mPus3p and mPus1p, whose specificity may depend on specific cellular contexts and which may influence whether SRA functions as coactivator or corepressor (71). U206 is located within the stem-loop substructure STR5 of the core conserved SRA [also defined as helix 7 within the newly characterized SRA full secondary structure (45)]. STR5 has been shown, at least in part, to contribute to the full activity of SRA (43). Interestingly, synthetic STR5 can inhibit Pus1p-mediated pseudouridylation of SRA, and has higher efficiency than mutant SRA-U206A to inhibit transactivation of ERα and AR. This suggests that STR5 could be a novel therapeutic target in control of NR transcriptional activity (55).

### SRA regulates the cell cycle

Lanz et al*. first* recognized the role of SRA in regulating cell proliferation via studies of mammary epithelial cells (68). We recently further revealed that SRA also promotes S-phase entry during mitotic clonal expansion of preadipocytes, which occurs shortly after the induction of adipogenesis. Here, SRA decreases expression of the cyclin-dependent kinase inhibitors p21Cip1 and p27kip1 and increases the phosphorylation of Cdk1 and Cdc2, suggesting that the ability of SRA to regulate the cell cycle may be independent of its coactivation of PPARγ (56). In addition, SRA knockdown reduces ligand-induced cell proliferation of T47D cells, and microarray analysis revealed that, among SRA-affected genes, there is enrichment for gene ontology (GO) terms related to apoptosis and cell proliferation (54) (Figure 2B). Thus, these observations demonstrate the ability of SRA to regulate the cell cycle across diverse cell types.

### Genome-wide SRA-responsive genes

Recently, three groups have reported the identification of genome-wide SRA-responsive genes in breast cancer cells or adipocytes. Foulds and colleagues used siRNAs to deplete SRA in HeLa cells and MCF-7 human breast cancer cells, followed by microarray analysis of the transcriptional consequences (76). They found that the majority of significantly changed genes are downregulated by SRA knockdown, consistent with SRA functioning as a coactivator. In HeLa cells, 4030 probe sets are differentially regulated following SRA knockdown. In MCF-7 cells, 780 transcripts are differentially regulated by SRA knockdown and E2 induction, although only a small subset of these genes are direct targets of ERα. This suggests that SRA is not impacting gene expression via modulation of ERα activity. Instead, GO analysis reveals that in HeLa cells SRA may play roles in protein targeting to membranes, transporting metal ions and lipids/cholesterol, as well as affecting fatty acid biosynthesis; while in E2-treated MCF-7 cells, GO data suggests possible SRA functions related to thyroid metabolism, such as reduced expression of DIO2.

Another study investigated the transcriptional consequences of SRA knockdown in the T47D breast cancer cell line, in comparison to PR target genes. Here, 2,343 transcripts were found to be regulated by treatment with R5020, a PR agonist, among which 466 transcripts (20%) were also affected by SRA knockdown (54). Interestingly, among these 466 SRA-responsive genes, 60% were up-regulated and 40% were down-regulated by SRA knockdown and R5020 treatment. GO analysis further indicated that SRA-responsive genes are involved in regulation of transcription and phosphorylation, cell proliferation, apoptosis, DNA binding, intracellular signaling cascades, and signal transduction. Thus, these roles may differ to those observed above for SRA-responsive genes in HeLa and MCF-7 cells.

The above transcriptomic analyses illustrate the diverse mechanisms through which SRA's transcriptional regulatory actions might impact cell biology. To further address this, we have recently identified SRA-responsive genes in SRA-overexpressing ST2 adipocytes and SRA knockdown 3T3-L1 adipocytes (56). In SRA-overexpressing ST2 adipocytes, 1687 genes/expressed-sequence tags (EST) are significantly changed. Among these, 421 (25%) were up-regulated and 1,266 (75%) were down-regulated by SRA overexpression. For 3T3-L1 adipocytes, the expression of 340 genes/ESTs was differentially regulated (145 up, 195 down) by SRA knockdown. In the ST2 adipocytes, GO analysis revealed that SRA overexpression is associated with enrichment of genes related to calcium ion binding, receptor activity, actin binding, molecular transducer activity, extracellular matrix structural constituents conferring tensile strength, and GTPase action. In 3T3-L1 adipocytes, SRA knockdown is associated with enrichment for genes involved in cell adhesion, phosphate transport, collagen fibril organization, transmembrane receptor protein tyrosine kinase signaling, axon extension, and multicellular organismal development. The mechanisms and functional implications of these diverse transcriptional effects remain to be fully elucidated. However, these observations highlight the diverse pathways through which SRA might impact physiology and disease. These pathways are further discussed below.

### SRA regulates multiple signaling pathways

As described above, in both cancer cells and adipocytes, SRA-responsive genes include those involved in transmembrane receptor-mediated signal transduction, suggesting that SRA may regulate pathways responsive to extracellular effectors (Figure 2B). Indeed, we found that SRA overexpression inhibits the expression of a subset of negative regulators of insulin sensitivity, such as SOCS-1/-3, Pkca, and Foxc2 (56). This is in agreement with the finding that SRA increases insulin-stimulated glucose uptake and phosphorylation of Akt and FOXO1 in mature adipocytes. In addition, gene set enrichment analysis (GSEA) identifies SRA-responsive and functionally related gene sets enriched in TNFα signaling. This finding was further confirmed by experiments showing that SRA inhibits TNFα-induced JNK phosphorylation. To assess the mechanism, our recent study further demonstrated that SRA overexpression inhibits the phosphorylation of p38 MAPK and JNK, while SRA knockdown increases JNK phosphorylation (44). Furthermore, knockdown of SRA in 3T3-L1 mature adipocytes led to decreased insulin receptor (IR) mRNA and protein levels, thereby leading to decreased autophosphorylation of IRβ and decreased phosphorylation of IR substrate-1 (IRS-1) and Akt. This effect likely reflects a stimulatory role of SRA on IR transcription (44). Recently, we have also shown that SRA enhances insulin signaling in hepatocytes (74). Taken together, these data suggest that SRA promotes adipocyte differentiation and insulin sensitivity through up-regulating insulin signaling and simultaneously inhibiting signaling downstream of TNFα, which otherwise would promote insulin resistance.

Interestingly, a recent study revealed that SRA associates with the RNA helicase Ddx5/p68 in murine Beko pre-T cells, and acts as a coactivator in the Notch signaling pathway (77) (Figure 2B). It remains possible that SRA might also modulate insulin, TNFα and/or Notch signaling pathways in cancer cells and other cell types, thereby regulating cell proliferation and differentiation; however, this possibility remains to be addressed.

### SRA in activating and repressive complexes

Most early studies, based on reporter gene assays, revealed that SRA functions as a scaffold to coactivate multiple transcriptional activities of NRs. The molecular mechanisms underlying this coactivator function have begun to be elucidated (Figure 3). Thus, SRA has been shown to form a complex with the p160 coactivator SRC-1, indicating that SRA may modulate the activity of NRs by forming a distinct activating complex (23, 78). Subsequent studies have revealed that SRA also can form a complex with the p68/p72 RNA helicases to coactivate MyoD transcription and stimulate expression of downstream target genes (62). In addition, we have shown that SRA binds to Dax-1 to coactivate transcriptional activity of SF-1 in steroidogenesis, and to augment liver receptor homolog 1 (LRH-1)-mediated Oct4 activation (59, 61). A recent study further demonstrated that SRA functions as a scaffold binding to the RNA-induced silencing complex (RISC) proteins PACT, TRBP, and Dicer to activate a subset of NRs, including ER, GR, and AR (72). However, SRA can also suppress NR transactivation by binding to nuclear repressors such as SHARP (the SMRT/HDAC1 associated repressor protein) (48) and SLIRP (SRA stem-loop interacting RNA binding protein) (47). These observations suggest that SRA also plays a repressive role for gene expression in some cellular contexts. Indeed, a recent study further revealed that SRA binds to PR, HP1γ (heterochromatin protein 1γ), and LSD1 (lysine-specific demethylase 1), and its depletion compromises the loading of the repressive complex to target chromatin-promoting aberrant gene de-repression. These data indicate that SRA exists in a repressive complex containing HP1γ, LSD1, HDAC1/2, CoREST (corepressor for REST [RE1-neuronal repressor element 1] silencing transcription factor), and demethylase KDM5B (54). In addition, unliganded PR binds genomic sites and targets this SRA-containing repressive complex to 20% of hormone-inducible genes, maintaining these genes in a silenced state prior to hormone treatment. Thus, in this class of target genes, SRA may play a repressive role (54) (Figure 3). As discussed below, we also recently showed that SRA inhibits forkhead box protein O1 (Foxo1) transcriptional activity in hepatocytes (74). Therefore, our current model suggests that SRA can exist in both coactivator and repressor complexes to either promote or inhibit transcriptional activity of NRs or other TFs. The switch to determine if SRA activates or inhibits gene expression may depend on the cellular context, for example in response to hormones or other specific physiological stimuli (Figure 3).

### SRA in chromatin modification and epigenetic regulation

Chromatin insulators are DNA elements that can protect a gene from outside influence, leading to either inappropriate activation or silencing of the gene (79, 80). CTCF is a DNA-binding protein that can control higher-order chromosomal looping and insulate specific genes from the effects of long-range enhancers and regulatory elements (81). Yao and colleagues recently revealed that SRA/p68 form a complex with CTCF that is essential for insulator function in HeLa cells (69). Knockdown of SRA or p68 reduced the CTCF-mediated insulator activity at IGF2/H19 ICR, increased the expression of *IGF2* and increased the interaction between the endodermal enhancer and the *IGF2* promoter. SRA also interacts with members of the cohesin-binding complex, and knockdown of SRA reduces cohesin binding although this does not affect CTCF genomic-binding sites.

Increasing evidences demonstrate that lncRNAs can target diverse chromatin regulators and several chromatin modifications (10, 16). Modification of histone 3 by lysine 4 trimethylation (H3K4me3) and lysine 27 trimethylation (H3K27me3) represent activating and repressive histone marks, respectively. When they present together as bivalent chromatin domains, they control the precise gene expression programs during pluripotency and differentiation that are poised for induction (82). There are two distinct histone modification machineries including complexes of the trithorax (TrxG) and polycomb repressive complex 2 (PRC2), which are responsible for methylation of H3K4 and H3K27, respectively. As shown in Figure 3, SRA binds to a subset of proteins belonging to both TRxG and PRC2, thereby regulating the formation of these complexes. One recent study indicated that, in mouse embryonic stem cells, SRA can interact with EZH2, a component of PRC2 (70). Similarly, in human pluripotent NTERA2 stem cells, SRA associates with TRxG components via specific interactions with WDR5, while in the PRC2 complex SRA directly binds to SUZ12 and EED (73). This study further revealed that SRA-associated p68 stabilizes SRA's interaction with TrxG, but not PRC2 (73). By using the technique of chromatin isolation by RNA purification (ChIRP) (83), 7,899 SRA genome-wide binding sites were identified to colocalize with bivalent domains for both H3K4me3 and H3K27me3 in NTERA2 stem cells (73). The same report also indicated that, in these stem cells, the transcription factor NANOG directly interacts and co-localizes with SRA on a genome-wide level, with this interaction being important for maintaining the stem cell pluripotent state. Although RNA-seq analysis for gene expression has not been reported in this study, these SRA-binding sites are likely the direct genome-wide targets that SRA regulates. These data suggest that, at least in stem cells, SRA likely regulates gene expression and cellular function by forming complexes with TrxG and PRC2 proteins, thereby establishing bivalent H3 methylation domains. This epigenetic mechanism may also recruit diverse DNA-binding factors that are able to activate or repress transcription in response to various cellular signaling pathways (Figure 3). However, it remains unknown if these epigenetic functions of SRA extend beyond stem cells. Thus, it will be crucial to investigate if SRA applies similar mechanisms to regulate the function of cancer cells and other cell types.

## SRA and human disease

The need to understand these mechanisms in the context of cancer is particularly important, because there are increasing evidences showing that SRA plays an important role in the development of breast and prostate cancers. However, SRA has also been implicated in the pathogenesis of other diseases (Figure 2A). Consequently, there could be great therapeutic benefit to better understanding the mechanisms through which SRA impacts cancer and other disease states.

### SRA and breast cancer

SRA expression is significantly up-regulated in human tumors of steroid-responsive tissues including the breast, uterus and ovary (50, 68). One study revealed that breast cancer cells generate both SRAP-coding and non-coding SRA isoforms by alternative splicing (29). Furthermore, in T5 breast cancer cells, increases in the ratio of non-coding SRA (intron-1-contiaing) to SRAP-coding transcripts resulted in elevated expression of genes associated with cancer cell invasion and tumor progression (51). In contrast, SRAP levels were significantly higher in ERα-positive and PR-positive breast cancer biopsies, identifying SRAP as a potential new prognostic marker for breast cancer patients (84). A recent study further indicated that overexpressing SRAP increases breast cancer cell motility (85).

Other data support a direct role for SRA that may be independent of ERα action. For example, depletion of SRA in two human breast cancer cell lines significantly reduced expression of the majority of SRA-responsive genes, suggesting a coactivating role of SRA (76). However, this study indicated that only a small subset of direct ERα-target genes was affected in estradiol-treated MCF-7 cells, in which SRA depletion led to decreased expression of some genes (such as THBS1, CAV1, TMPRSS2, and TMPRSS3) tightly associated with invasion/metastasis. Furthermore, SRA depletion in ERα-negative MDA-MB-231 cells decreased the expression of some genes important for invasiveness (such as CAV1, TMPRSS2, and 12 MMPs), and this was reflected in decreased invasive behavior of the SRA-depleted cells (76). In general, these experiments suggest that the effect of SRA on breast cancer may be less ER dependent than initially expected. In contrast, a recent case study evaluated the association between breast cancer risk and two haplotype tagging SNPs (htSNPs) (rs10463297, rs801460) within the *SRA1* locus (86). This case study indicated that these SRA htSNPs are significantly associated with ER positivity status and this may contribute to breast cancer risk and progression.

### SRA and prostate cancer

Recent studies indicate that SRA and other lncRNAs, such as PCA3, PCAT-1, and SCHLAP1, are involved in the progression of prostate cancer (87–91). For SRA this relationship may relate to effects of both the lncRNA and SRAP. For example, three SRAP isoforms are expressed in human prostate cell lines (34), and transfection of SRAP-coding SRAs promotes AR transcriptional activity in several prostate cancer cells (such as PC-3, DU-145, and LNCaP cells) (92). Conversely, knockdown of SRA inhibits AR activity in LNCaP and PC-3 cells (92). These data support the conclusion that SRA and SRAP influence prostate cancer by coactivating the AR. Consistent with this, in AR-positive LNCaP cells, knockdown of SRA results in reduced proliferation and decreased expression of *TMPRSS2*, but not *PSA* and *PMEPA1* (93). However, SRA knockdown also exerts these effects in DU145 cells, which are AR-negative (93), suggesting that SRA also exerts AR-independent effects to modulate prostate cancer progression. Whether these AR-independent effects are mediated via SRA or SRAP remains to be determined. Indeed, since the knockdown of the *SRA1* gene depletes both SRA and SRAP, future experiments are required to distinguish the functions of SRA and SRAP in the pathogenesis of prostate cancer.

### SRA, obesity, and fatty liver

Lanz and colleagues have shown that SRA expression is enriched in human liver and muscle tissues (23), key organs in the regulation of metabolic homeostasis. Interestingly, the previously described MMTV-SRA transgenic mice not only display a phenotype of increased cellular proliferation, differentiation, and ductal-epithelial hyperplasia; they also present with pronounced multilocular fat, including an increased density of fat droplets in mammary glands of adolescent MMTV-SRA mice (68). Consistent with this putative role of SRA in regulating adipose development *in vivo*, we recently revealed that, in wild-type mice, SRA is expressed most highly in adipose tissues (42). To directly assess the role of SRA in adipose tissue development, we generated the first mouse model with a whole-body knockout of SRA (SRA^−/−^). We have found that SRA^−/−^ mice are resistant to high-fat diet (HFD)-induced obesity, with decreased fat mass and increased lean mass (42). This lean phenotype of SRA^−/−^ mice is associated with decreased expression of a subset of adipocyte marker genes in adipose tissues, as well as reduced plasma TNFα levels. The latter is notable given that obesity is associated with increased production of TNFa within adipose tissue, which may contribute to metabolic dysregulation. Indeed, the SRA^−/−^ mice are more insulin sensitive than their wild-type counterparts; have reduced fatty liver after HFD feeding; and have decreased expression of hepatic lipogenesis-associated genes. Adipose triglyceride lipase (ATGL) is a major triacylglycerol (TAG) hydrolase that acts as a key regulator of hepatic lipid metabolism. Our recent study demonstrated that loss of SRA in hepatocytes upregulates ATGL expression and the β-oxidation of free fatty acids (FFA), whereas forced expression of SRA inhibits these outcomes (74). Furthermore, SRA represses ATGL expression primarily by inhibiting the inductive effects of Foxo1. Along with the previously discussed effects of SRA on adipogenesis and adipocytes *in vitro*, these data suggest that SRA may be a potential target to control HFD-induced obesity, fatty liver and glucose homeostasis *in vivo. One* key remaining question is whether SRA also impacts metabolic health in humans. For example, are SNPs in *SRA1* associated with altered adiposity, hepatic lipid content and/or other metabolic outcomes? Such genome-wide human studies will be important to pursue if we are to better understand the interplay between SRA and metabolic homeostasis in humans.

### SRA and cardiovascular disease

In a recent genetic study to identify genes responsible for the pathogenesis of human dilated cardiomyopathy, SRA emerged as one of the key candidate genes (94). To functionally address this possibility, the authors treated fertilized zebrafish eggs with antisense morpholinos against SRA, thereby decreasing SRA expression. This resulted in a phenotype of myocardial contractile dysfunction with impaired contractility, predominantly in ventricular heart chambers at the early stage of heart development (94). Previous studies have established roles for the thyroid hormone and glucocorticoid receptors in cardiac function (95, 96); hence, given the ability of SRA to impact NR action, one possibility is that depletion of SRA leads to defective heart development as a result of decreased activity of these NRs. However, this possibility remains speculative, and the mechanisms linking SRA to cardiovascular function in zebrafish remain to be elucidated. More importantly, future studies must address if SRA also impacts cardiovascular health in higher organisms, including functional studies in mammalian systems and genetic studies in human populations.

### SRA and diseases of reproductive health

Polycystic ovary syndrome (PCOS) is one of the most common heterogeneous endocrine and metabolic disorders. PCOS accounts for 75% of anovulatory infertility and is highly associated with obesity, insulin resistance, dyslipidemia and increased risk of diabetes and cardiovascular events (97). A recent case study revealed that SRA expression in peripheral blood leukocytes was significantly higher in women with PCOS than in healthy control individuals (28). However, at this stage it remains unknown if altered SRA expression contributes to the pathogenesis of PCOS, highlighting this topic as another area for future investigation.

Other recent case studies have identified three independent families with inactivating SRA1 mutant variants (missense mutations including P20L, Q32E, Y35N, or I179T) that are associated with delayed puberty/normosmic idiopathic hypogonadotroic hypogonadism (IHH) (25). This disease is characterized by an inability to develop secondary sexual characteristics and a mature reproductive system, owing to defects in the central part of the hypothalamic-pituitary-gonadal axis (98). These case studies suggest that SRA1 gene function may be required for the initiation of puberty in humans.

In addition to PCOS and IHH, two final studies implicate SRA in the pathogenesis of endometriosis, an estrogen-dependent disease that affects about 10% of reproductive-aged women. Interestingly, Lin and colleagues reported that SRA RNA and ERα protein expression are higher in endometrial tissue compared to ovarian tissue surrounding mature teratoma and ovarian endometrioma (27). In contrast, higher protein expression of SRAP and ERβ were shown in surrounding ovarian tissue than in ovarian endometrioma and ovarian tissue surrounding mature teratoma. In human primary endometriotic stromal cells, knockdown of the SRA gene increases ERα but decreases ERβ expression, as well as attenuates cell proliferation and promotes early apoptosis when cells are treated with E2 (99). These observations suggest that SRA/SRAP may act in concert to regulate the function of ERα and ERβ in the pathogenesis of ovarian endometrioma.

In summary, studies relating to the pathogenic roles of SRA have focused largely on breast and prostate cancers, as well as metabolic diseases such as obesity-related insulin resistance and hepatic steatosis. Other studies are beginning to highlight potential roles of SRA in other diseases, including cardiovascular disease and reproductive disorders such as PCOS, IHH, and endometriosis. Future studies must now build on these promising findings, in particular through human analyses, so that we can better understand the pathogenic roles of SRA.

## Future questions and therapeutic implications

It has been almost two decades since the discovery of the lncRNA SRA. However, many aspects of SRA biology remain to be elucidated. For example, what is its exact physiological function? Through what molecular mechanisms does SRA regulate gene expression, development, and cell differentiation? Finally, how does SRA impact the pathogenesis of cancers and other diseases?

In terms of molecular mechanisms, we now know that SRA exists in the ribonucleoprotein complex that includes SRC-1 and binds to components of proteins belonging to both the TrxG and PRC2 complexes, which regulate chromatin's epigenetic state. SRA also binds to numerous protein partners including NRs and other transcription factors and coregulators. Thus, it is crucial to further identify the dynamic SRA-binding proteins or RNAs in living cells, including how these interactions are influenced by hormones or other physiological conditions. ChIRP is one approach that has much promise in revealing this new knowledge. In addition, how SRA-binding sites are regulated by different extracellular signals, in a cell-context dependent manner, is largely unknown. Therefore, it is necessary to identify SRA genome-wide binding sites and analyze their association with gene expression under specific differential conditions, developmental stages and in different cell types, such as during adipogenesis or breast cancer cell oncogenesis. This knowledge would improve our understanding of SRA's transcriptional targets and how these impact physiological and pathological processes.

A related question is, how is SRA recruited to chromatin? Clearly, this recruitment may be via known SRA-interacting partners, including NRs, other transcription factors (such as MyoD), coregualators (p68), and components of chromatin regulatory complexes (LSD1, TrxG, and PRC). However, other lncRNAs employ more diverse mechanisms, including the formation of RNA:DNA:DNA triplexes; generation of RNA:DNA hybrids that displace a single strand of DNA; or creation of an RNA:RNA hybrid between a lncRNA and a nascent transcript (10, 16). Does SRA also utilize any of these mechanisms to modulate gene expression? The ability of lncRNAs to bind to protein partners embues them with diverse regulatory properties. For example, it is possible that SRA plays a role through “decoys” that preclude the access of regulatory proteins to DNA, and/or through “guides” that facilitate correct localization of specific protein complexes (16). Clearly, these possibilities must be investigated in future studies.

Increasing evidences indicate that SRA can both activate and repress gene expression (Figure 3). Although recent studies indicate that SRA binds to components of both activating (TrxG) and repressive (LSD1 and PRC2) complexes, the precise mechanism involved is yet to be determined. Thus, it remains unclear if these interactions are further regulated, for example in response to stimuli related to different temporal and spatial aspects of development and cell differentiation. In addition, SRA also exists in other activating complexes (SRC1, p68, TIF2/Dax-1) and repressing complexes (SHARP and SLIRP/HDAC); how SRA works in concert with all these transcriptional coregulators and components of chromatin regulatory complexes (TrxG, LSD1, and PRC2) must be a focus of future research (Figure 3).

We and others have shown that SRA regulates insulin, TNFα and Notch signaling pathways, but many questions remain. For example, what is the molecular mechanism underlying this regulation? What are the upstream direct targets (transcription factors or signaling proteins) that regulate SRA function in response to these signaling pathways?

To determine the precise physiological function of SRA, it is crucial to establish the phenotypes of animal models of SRA gain- and loss-of-function, as well as the underlying mechanisms. Currently, neither transgenic MMTV-SRA mice (68) nor our SRA-/- mouse model can fully address this issue, because MMTV-SRA is expressed only in steroidal tissues and SRA^−/−^ mice lose both SRA and SRAP in all tissues (42). Future combined approaches could use CRISPR-Cas9 (100) to restore SRA or SRAP in ectopic sites (101, 102) under the SRA^−/−^ genetic background, or to delete endogenous SRA or SRAP on a tissue-by-tissue basis. Such highly specific approaches could more robustly address the physiological functions of SRA and SRAP in a tissue-specific manner.

Differential splicing of SRA must also be more closely investigated. As discussed above, some breast cancer tumor cells have high expression of intron-1-containg SRA, relative to the SRAP-encoding variants. Future studies must further address whether this pattern of elevated intron-1-containg SRA (relative to fully spliced SRA or other non-SRAP-coding SRA isoforms) occurs in other developmental or pathological contexts. For example, is the intron-1-containing SRA also enriched in the adipose tissue or liver of obese individuals, compared to healthy non-obese controls? These splicing processes are regulated by trans-acting proteins (repressors or activators) and corresponding cis-acting sites in the DNA promoter sequence of the *SRA1* gene. How these elements form a “splicing code” to regulate splicing under different physiological conditions (103, 104) is yet another subject for future study. Furthermore, what upstream regulatory mechanisms underlie the increased SRA expression in breast cancer cells, during adipocyte differentiation, and in fatty liver? Similarly, how is SRA expression regulated in response to the external stimuli and specific signaling pathways? These questions remain to be answered.

LncRNAs have been emerging as factors that provide a new layer for regulation of gene expression in numerous biological contexts, including cell differentiation, development, physiology, and disease. In addition to being a potential biomarker for breast cancer, the differential expression of SRA and genetic mutations have been connected to other diseases such as POCS, IHH, endometriosis, and cardiovascular dysregulation. However, only a handful of studies have reported a role for SRA in these contexts, underscoring the need for additional research.

### SRA as a novel therapeutic target

Experimental therapeutic agents targeting lncRNAs are only in their infancy. The characterization of the precise secondary structures of SRA promises to open new strategies. For example, it might be possible to target specific stem loop structures of SRA to more precisely modulate activities of nuclear receptors (55) and other transcription factors, and/or to impact epigenetic effects in the pathogenesis of SRA-related diseases. This improved understanding of SRA structure may also facilitate rational design of more precise SRA antisense oligonucleotides and/or other forms of knockdown methods that will allow finer modulation of SRA function. The aforementioned trans-acting proteins and corresponding cis-acting sites that are involved in regulating SRA splicing are also potential targets for controlling the production of disease-causing isoforms of SRA, such as the non-SRAP-coding intron-1-containing variant. This is exemplified by studies targeting regulatory elements in the H19 gene as an approach for pancreatic cancer therapy (105). Furthermore, more recent studies using engineered CRISPR-interference (CRISPRi) or CRISPR-activation (CRISPRa) can control gene expression at target promoters (106, 107). For example, two recent studies successfully proved that the promoter sequence of lncRNAs can be used as a target to regulate the gene expression and splicing of themselves and their neighboring genes (108, 109). Therefore, gene editing via CRISPR-Cas9 might allow tissue-specific manipulation of SRA expression as an improved precision-medicine approach.

## Concluding remarks

Much progress has been made in understanding the physiological and pathological functions of SRA, and some light has been shed on the underlying molecular mechanisms. However, from this review it is clear that many, many questions remain to be answered. Nevertheless, SRA and other lncRNAs display promise as biomarkers and therapeutic targets that might allow new strategies for improved clinical outcomes. To realize this promise, it is essential that future research yields further insights into the physiological and pathological roles of SRA, as well as the molecular mechanisms of its action.

## Author contributions

LS, LY, DZ, and BX wrote the manuscript, and BX designed figures. WC and BX revised the manuscript, and WC rewrote several sections of the manuscript.

### Conflict of interest statement

The authors declare that the research was conducted in the absence of any commercial or financial relationships that could be construed as a potential conflict of interest.
